# Accelerating Whole-Body Diffusion-weighted MRI with Deep Learning–based Denoising Image Filters

**DOI:** 10.1148/ryai.2021200279

**Published:** 2021-07-14

**Authors:** Konstantinos Zormpas-Petridis, Nina Tunariu, Andra Curcean, Christina Messiou, Sebastian Curcean, David J. Collins, Julie C. Hughes, Yann Jamin, Dow-Mu Koh, Matthew D. Blackledge

**Affiliations:** From the Division of Radiation Therapy and Imaging, The Institute of Cancer Research, 123 Old Brompton Rd, London SW7 3RP, England (K.Z.P., N.T., A.C., C.M., S.C., D.J.C., J.C.H., Y.J., D.M.K., M.D.B.); and Department of Radiology, The Royal Marsden National Health Service Foundation Trust, Surrey, England (N.T., A.C., C.M., S.C., J.C.H., D.M.K.).

**Keywords:** Image Postprocessing, MR-Diffusion-weighted Imaging, Neural Networks, Oncology, Whole-Body Imaging, Supervised Learning, MR-Functional Imaging, Metastases, Prostate, Lung

## Abstract

**Purpose:**

To use deep learning to improve the image quality of subsampled images (number of acquisitions = 1 [NOA_1_]) to reduce whole-body diffusion-weighted MRI (WBDWI) acquisition times.

**Materials and Methods:**

Both retrospective and prospective patient groups were used to develop a deep learning–based denoising image filter (DNIF) model. For initial model training and validation, 17 patients with metastatic prostate cancer with acquired WBDWI NOA_1_ and NOA_9_ images (acquisition period, 2015–2017) were retrospectively included. An additional 22 prospective patients with advanced prostate cancer, myeloma, and advanced breast cancer were used for model testing (2019), and the radiologic quality of DNIF-processed NOA_1_ (NOA_1-DNIF_) images were compared with NOA_1_ images and clinical NOA_16_ images by using a three-point Likert scale (good, average, or poor; statistical significance was calculated by using a Wilcoxon signed ranked test). The model was also retrained and tested in 28 patients with malignant pleural mesothelioma (MPM) who underwent lung MRI (2015–2017) to demonstrate feasibility in other body regions.

**Results:**

The model visually improved the quality of NOA_1_ images in all test patients, with the majority of NOA_1-DNIF_ and NOA_16_ images being graded as either “average” or “good” across all image-quality criteria. From validation data, the mean apparent diffusion coefficient (ADC) values within NOA_1-DNIF_ images of bone disease deviated from those within NOA_9_ images by an average of 1.9% (range, 1.1%–2.6%). The model was also successfully applied in the context of MPM; the mean ADCs from NOA_1-DNIF_ images of MPM deviated from those measured by using clinical-standard images (NOA_12_) by 3.7% (range, 0.2%–10.6%).

**Conclusion:**

Clinical-standard images were generated from subsampled images by using a DNIF.

**Keywords:** Image Postprocessing, MR-Diffusion-weighted Imaging, Neural Networks, Oncology, Whole-Body Imaging, Supervised Learning, MR-Functional Imaging, Metastases, Prostate, Lung

*Supplemental material is available for this article.*

Published under a CC BY 4.0 license.

## Graphical abstract

**Figure:**
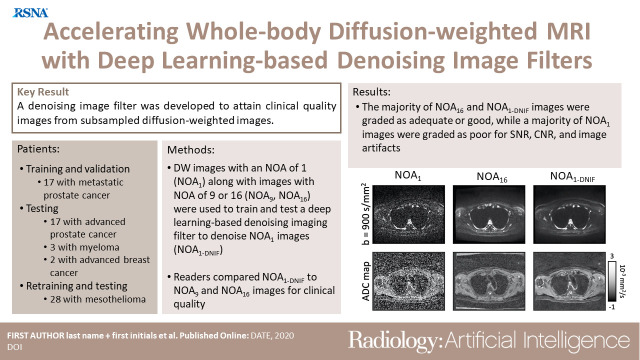

**Summary**

A developed model, called quickDWI, enabled accelerated acquisition protocols for whole-body diffusion-weighted MRI of metastatic prostate, breast, and myeloma bone disease by using deep learning, resulting in images that were comparable with clinical-standard images.

**Key Points**

■ A U-Net–based architecture can successfully reduce the magnitude of noise present in diffusion-weighted MR images; the average mean absolute error of all validation images acquired at *b* values of 50, 600, and 900 sec/mm^2^ was reduced from 0.87 × 10^−3^ to 0.53 × 10^−3^.
■ The algorithm significantly improved the radiologic image quality of fast but noisy whole-body MRI data in 22 patients with bone disease (*P* < .01).
■ The algorithm could reduce whole-body diffusion-weighted MRI times from 25–30 minutes to approximately 5 minutes.

## Introduction

Whole-body diffusion-weighted MRI (WBDWI) is a noninvasive tool used for staging and response evaluation in oncologic practice and is at the core of emerging response criteria in advanced prostate and breast cancers (1–4). WBDWI has recently been incorporated into the National Institute for Health and Care Excellence guidelines for assessing myeloma-related bone disease (5,6). Through its sensitivity to water diffusion within tissue, WBDWI is a sensitive tool that radiologists can use to review the extent of disease within the skeleton. Moreover, use of WBDWI enables the voxel-wise quantification of the change in the apparent diffusion coefficient (ADC), providing a potential marker for tumor response assessment (7).

WBDWI is typically performed through a series of sequential imaging stations from the head to the midthigh, with each station consisting of 30–50 axial sections, with images acquired by using two to three diffusion weightings (1,8). Therefore, WBDWI accounts for more than 50% of the acquisition time of conventional whole-body MRI studies with a 1-hour duration. In the context of the ever-increasing capacity pressures on MRI departments, reducing acquisition times would facilitate the wider adoption of clinical WBDWI, reduce costs, and improve the patient experience (9,10).

In this proof-of-concept study, we hypothesized that the use of U-Net deep learning architectures could allow fivefold to 10-fold reduction in imaging times by recovering fully sampled WBDWI images with a high signal-to-noise ratio (SNR) from undersampled images with a low SNR. U-Net–inspired architectures (11) can contextualize image features at multiple spatial resolutions and then upsample them to increase the resolution of the output. Applications include automatic segmentation (12), lesion classification (13), image reconstruction (14), quantitative susceptibility mapping (15), artifact reduction (16), and image denoising (17,18). We trained our model on a sample of patients with advanced prostate cancer and subsequently tested it on a separate prospective sample of patients with advanced prostate cancer, advanced breast cancer, and myeloma. In addition, to test the feasibility of the technique for diffusion-weighted MRI (DWI) acquisitions obtained over a smaller field of view, we retrospectively analyzed a sample of patients with malignant pleural mesothelioma (MPM) (19).

## Materials and Methods

### Patient Population and Imaging

These studies were reviewed and approved by our local research ethics committee. The ethics committee waived the requirement of written informed consent for participation.

**Figure 1:**
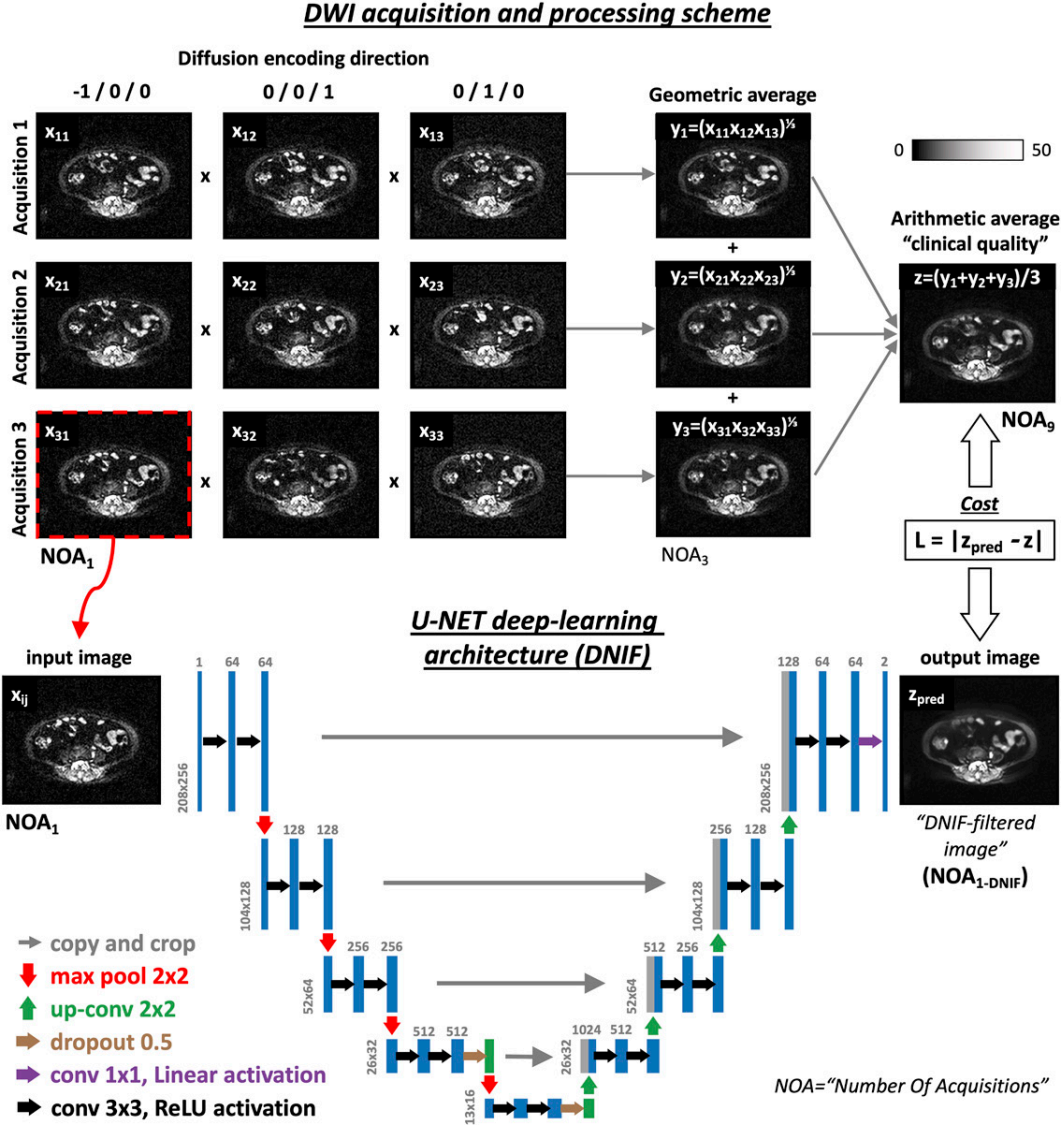
(Top) Generation of “clinical-standard” images, *z* (number of acquisitions = 9 [NOA_9_]), from the single acquisition images, *xij* (NOA_1_), is achieved by computing the geometric average over the different directions, *j*, and computing the arithmetic average over the resulting trace-weighted images, *yi* (NOA_3_). Such operations mimic the processing performed by most clinical imagers when acquiring whole-body diffusion-weighted MR images. In clinical images, only the averaged images (*z*) are retained, whereas all other data are removed to reduce storage requirements. (Bottom) Our deep learning–based denoising image filter’s (DNIF’s) U-Net–like architecture for processing the input of noisy diffusion-weighted images from a single acquisition (NOA_1_) at a random *b*-value direction, *x1j*, to predict image *z* (DNIF-predicted *z* [*z*_pred_]) is shown. The network extracts multiscale features from the NOA_1_ image and subsequently reconstructs the image by using the acquired clinical-standard (NOA_9_) image as the ground truth. The mean absolute error (loss [*L*]) is used as the cost function to evaluate the perceived closeness of *z*_pred_ to the acquired clinical-standard (ie, ground truth or NOA_9_) image, *z*. Apparent diffusion coefficient (ADC) maps derived from DNIF-processed images are calculated as a subsequent step by using a least-squares fitting approach after the individual denoised *b*-value images are estimated by using our model. conv = convolution, DWI = diffusion-weighted MRI, ReLU = rectified linear unit.

***Training WBDWI dataset.—*** WBDWI was performed with a 1.5-T Siemens Aera system at three *b* values (50, 600, and 900 sec/mm^2^) (3) in 17 men with suspected advanced prostate cancer over four to five axial imaging stations (October 2015 to September 2017; parameters are presented in Table 1). This retrospective sample included consecutive patients (age range, 49–82 years) with metastatic prostate cancer that required clinical evaluation of known metastatic bone disease by using WBDWI. For each section position, images were acquired at three different *b* values, at three orthogonal diffusion-encoding directions without averaging, and the individual direction images were retained (number of acquisitions = 1 [NOA_1_]). This acquisition was repeated three times, and a “trace-weighted” image (NOA_9_) was computed for each *b* value to derive the clinical-quality images (method illustrated in Fig 1). Data were randomly split into training (*n* = 14) and validation (*n* = 3) sets. These data were used in a previous publication investigating the utility of multiple image acquisitions (NOA_1_) for estimating whole-body ADCs through weighted least-squares approximation, along with voxel-wise characterization of the uncertainty in the derived ADCs (21).

**Table 1:**
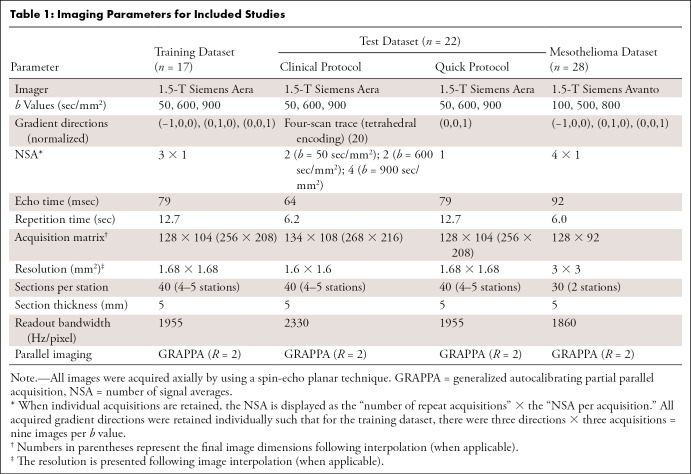
Imaging Parameters for Included Studies

***Test WBDWI dataset.—***WBDWI data were prospectively acquired after the acquisition of the training WBDWI dataset over a 2-month period (May and June 2019) in a separate sample of 22 consecutive patients with advanced prostate cancer (*n* = 17, all men), myeloma (*n* = 3, all men), and advanced breast cancer (*n* = 2, all women) who required clinical evaluation for suspected metastatic disease (age range, 39–84 years). Inclusion criteria were any patient undergoing whole-body MRI for clinical management of secondary bone disease who was deemed fit by the referring radiologist for an additional 5 minutes of imaging time; the exclusion criteria were any contraindication to MRI, including patient claustrophobia. For each patient, images were acquired by using two WBDWI protocols within the same study (the patient remained on the couch between protocols): the first protocol was the same as that performed for the training dataset, except that only a single acquisition at a single diffusion-encoding direction (NOA_1_) was obtained; the second protocol was an institutional clinical protocol (NOA_16_; parameters are presented in Table 1). The approximate acquisition times for these protocols were 5 minutes and 22–25 minutes, respectively. Patients also underwent whole-body Dixon imaging and sagittal T1-weighted and T2-weighted anatomic spine imaging as per standard clinical care (1,8). Images were acquired by using a 1.5-T Siemens Aera system.

***Mesothelioma dataset.—***To demonstrate the feasibility of our approach for smaller field-of-view imaging, we retrospectively evaluated data from a sample of 28 patients (four women and 24 men; age range, 52–85 years) imaged for the presence of MPM as part of a single-center study investigating the value of DWI in MPM (February 2015 to November 2017). Patients underwent lung MRI with a 1.5-T Siemens Avanto system. Imaging parameters are provided in Table 1; data were randomly split into a training dataset of 20 and a validation dataset of eight.

### Deep Learning Architecture

We developed our quickDWI method by training a deep learning–based denoising image filter (DNIF) model to generate clinical-grade diffusion-weighted images (NOA_9_) from images acquired by using one diffusion-encoding direction and one signal average with *b* values of 50, 600, or 900 sec/mm^2^ independently (DNIF-processed NOA_1_ [NOA_1-DNIF_] images; original acquired images are referred to as NOA_1_ images), as illustrated in Figure 1. For this purpose, we adapted a convolutional neural network based on the U-Net architecture (11), which has been modified to solve regression problems. A NOA_1_ image of 256 × 208 pixels in size was provided as input into the network (postlinear interpolation) and was grayscale normalized from a range of 0–4095 to a range of 0–1. After empiric experimentation, a linear activation was used for the last layer, whereas a rectified linear unit activation function was used in all preceding layers. We constrained the weights incident to each hidden unit to have a norm value of less than or equal to 3, the weights of the layers were randomly initialized by using He normal initialization (22), and the network was trained with a batch size of 36 for 15 epochs and optimized by using the Adam algorithm (23) with a learning rate of 0.001. The network was trained by using a Tesla P100 PCIE, 16-GB graphics processing unit card (Nvidia), and the trained algorithm was applied by using a MacBook Pro laptop (Apple) with a 2.9-GHz Intel Core i7 central processing unit (16-GB–2133-MHz random access memory with a low-power double data rate of 3).

We experimented with three cost functions that measured the similarity between the NOA_1-DNIF_ images and the clinical-grade (NOA_9_ and NOA_12_) images used as the ground truth: the mean-squared error (MSE) (24), the mean absolute error (MAE), and a combination of the MAE and the structural similarity (SSIM) index (25): 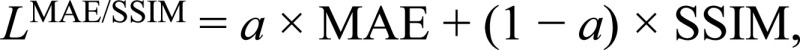 where *a* is the weight of each loss function and *L*^MAE/SSIM^ is the combined loss. We empirically set *a* to 0.7 after experimentation with different values.

The training WBDWI dataset provided a total of 59 400 training images (14 patients × three directions × three acquisitions × three *b* values) × [(80 sections × one patient with acquisition at only abdomen or pelvis stations) + (160 sections × 12 patients) + (200 sections × one patient)]. This dataset also provided a total 15 120 validation images (three patients). The mesothelioma dataset provided 43 200 training images (20 patients) and 15 120 validation images (eight patients). The images were normalized from a range of 0–939 to a range of 0–1 prior to input into the model. All code was written in Python (version 3.6.5.) by using the Keras and/or TensorFlow libraries.

### Data Analysis

***Training WBDWI dataset.—***As a measure of similarity to the NOA_9_ images, the MSE, SSIM, and peak SNR (PSNR) were computed for the NOA_1-DNIF_ and NOA_1_ images across all *b*-value images from all three validation patients (calculated by using scikit-learn version 0.14.2). A monoexponential, least-squares fitting algorithm was used to calculate ADC maps by using data from all three *b* values for the NOA_1_, NOA_9_, and NOA_1-DNIF_ images. A radiologist delineated regions of bone disease on the NOA_9_ images by using an in-house semiautomatic segmentation tool for WBDWI studies of advanced prostate cancer (26) for all validation patient images, and the resulting regions of interest were copied onto the derived ADC maps. The mean ADCs within regions of bone disease were compared across the three imaging schemes by calculating the relative difference of means (RDM) between NOA_1-DNIF_ or NOA_1_ ADC maps and NOA_9_ ADC maps: 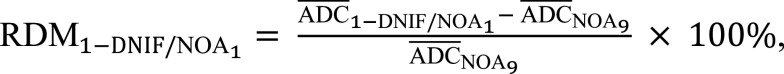 where ADC_1-DNIF/NOA1_ represents the mean ADC within the defined regions of interest for the NOA_1-DNIF_ or NOA_1_ images, respectively. Furthermore, we calculated the coefficient of variation as the standard deviation divided by the average ADC, and the mean absolute voxel-wise difference between the NOA_1-DNIF_ or NOA_1_ ADC maps and the NOA_9_ ADC maps. The distributions of ADC measurements within disease were compared for all methods by using violin plots; negative calculated ADCs were included in this analysis because they convey important information regarding the distribution of imaging noise.

***Test WBDWI dataset.—***The DNIF was directly applied to the test WBDWI dataset without further retraining. Two radiologists with 1 year (A.C.) and 10 years (D.M.K.) of experience with using WBDWI for the assessment of metastatic disease reviewed the NOA_16_, NOA_1_, and NOA_1-DNIF_ images of all 22 patients (readers were blinded to patient clinical details, and images were presented in random order). In each case, radiologists had access to all *b*-value images (50, 600, and 900 sec/mm^2^), and the ADC maps were calculated offline by using a monoexponential, least-squares fitting algorithm. Anatomic images were not provided to ensure a blinded reading. The radiologists qualitatively scored the contrast-to-noise ratio, SNR, and image artifacts of the *b* = 900 sec/mm^2^ images and the ADC maps independently by using a three-point Likert scale (1 = poor, 2 = adequate, and 3 = good). To assist in the qualitative assessment of the SNR and contrast-to-noise ratio metrics, the radiologists reported the average pixel values within regions of interest around a single site of disease surrounded by healthy tissue and background air on *b* = 900 sec/mm^2^ images.

***Mesothelioma dataset.—***We compared two versions of the DNIF model: a version incorporating direct application of the WBDWI dataset model without updating of the model parameters (WBDWI model) and a version that was retrained from scratch with 20 of the patients with MPM (lung model). The MSE, SSIM, and PSNR scores were calculated for all eight validation patients, as they had been for the training WBDWI dataset. Regions of disease were delineated on axial *b* = 50 sec/mm^2^ images for all eight validation patients by using 3D Slicer (27) and were then copied onto ADC maps calculated from NOA_12_, NOA_1_, and NOA_1-DNIF_ images. The mean ADCs within disease were compared across all four imaging schemes by using the same RDM, coefficient of variation, and mean absolute voxel-wise difference scores that were used for the training WBDWI dataset; ADC distributions were compared by using violin plots (including negative ADC values).

### Statistical Analysis

For the test WBDWI dataset, we calculated the statistical significance of differences between radiologist ratings of image quality for NOA_1-DNIF_ compared with NOA_1_ images and for NOA_12_ images compared with NOA_1_ images by using a Wilcoxon signed rank test. Comparisons were made for each image-quality metric, each observer, and for *b* = 900 sec/mm^2^ images and ADC maps independently. We used the “wilcoxon” function in the SciPy Python package (version 1.2.1) to perform our evaluations, assuming a two-sided alternative hypothesis. Calculated *P* values were corrected for multiple comparisons by using the Benjamini-Hochberg procedure, and a *P* value of less than .05 was chosen to indicate significance.

## Results

### Performance of the Deep Learning Network

Within 15 epochs, the network minimized the MAE, resulting in a change from 0.87 × 10^−3^ to 0.53 × 10^−3^, and minimized the *L*^MAE/SSIM^ metric, resulting in a change from 0.39 × 10^−2^ to 0.11 × 10^−2^. Both cost functions resulted in the same MAE solution (0.53 × 10^−3^). Interestingly, the network reached a better solution for the MSE through using either the MAE cost function or the *L*^MAE*/*SSIM^ cost function than through trying to minimize the MSE directly (MSE from MAE: 1.89 × 10^−6^ vs MSE from *L*^MAE*/*SSIM^ 1.88 × 10^−6^ vs direct MSE: 2.7 × 10^−6^). Through visual inspection of the training WBDWI dataset, an expert radiologist (N.T., 10+ years of experience) concluded that the network trained on the MSE cost function resulted in oversmoothing of the images without preserving edges, and so we used the MAE in all further training.

The network required 8 hours of training on the WBDWI data when using a Tesla P100 for PCIE 16-GB graphics processing unit card. In terms of computational efficiency, the trained network requires approximately 1 second to process a single low-SNR image on our MacBook Pro laptop with a 2.9-GHz Intel Core i7 central processing unit (16-GB–2133-MHz random access memory with a low-power double data rate of 3).

### Model Performance on the Validation WBDWI Sample

**Figure 2:**
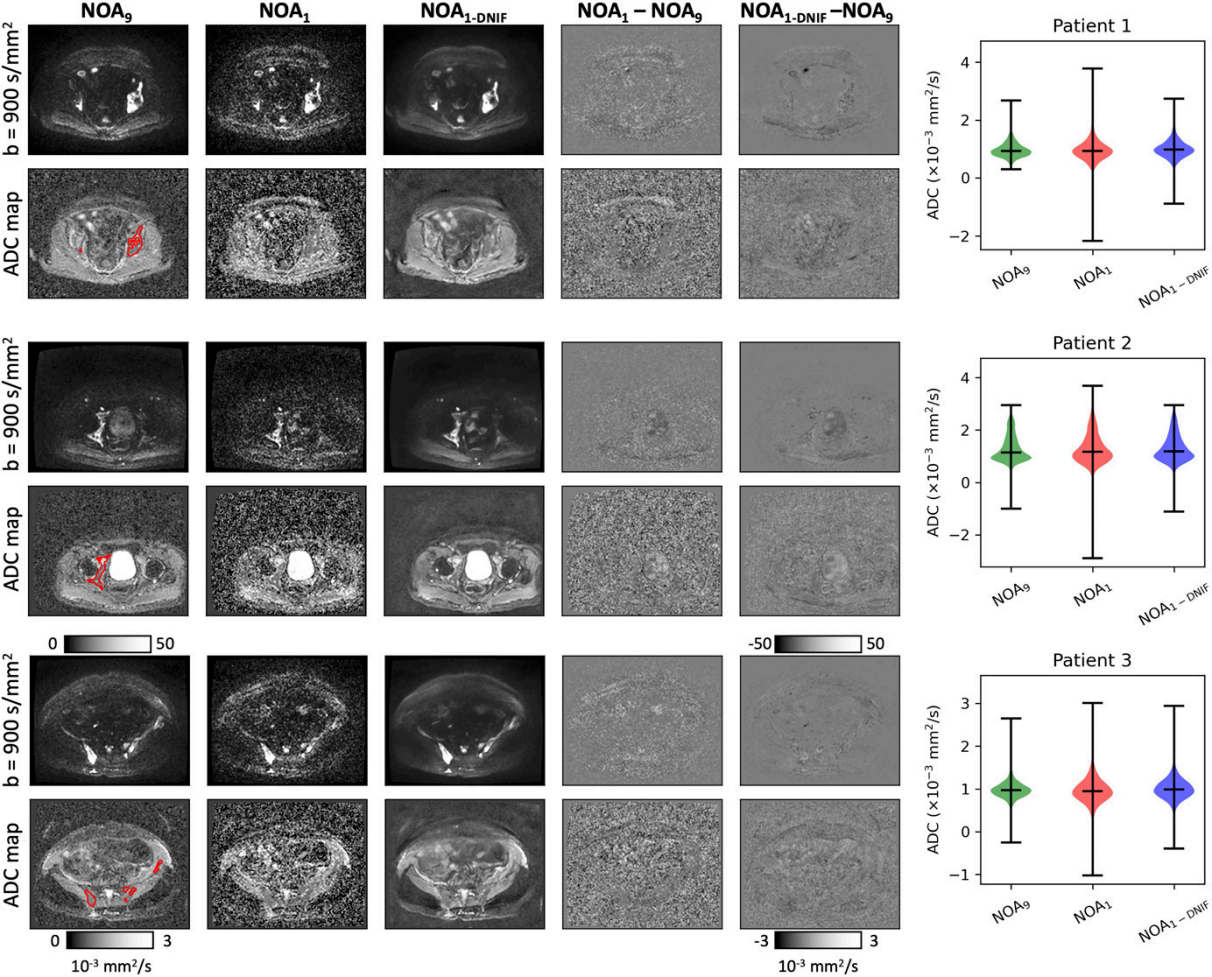
(Left) Example images from each of the three validation patients in the training whole-body diffusion-weighted MRI dataset. High-*b*-value images (*b* = 900 sec/mm^2^: top row in each patient example) are displayed alongside apparent diffusion coefficient (ADC) maps (bottom row in each patient example) for the clinical-standard (number of acquisitions = 9 [NOA_9_]) images, the fast-acquisition images (NOA_1_), and the deep learning–based denoising image filter (DNIF)–processed NOA_1_ (NOA_1-DNIF_) images. In addition, difference maps are shown between the clinical-standard images and the NOA_1-DNIF_ or NOA_1_ images (NOA_1-DNIF_ − NOA_9_, for example). All equivalent images are displayed by using the same windowing settings. (Right) Violin plots of the ADC distributions within segmented bone disease for the same three patients (example segmentation regions are displayed as red contours on NOA_9_ ADC maps). It is clear that there is a reduction in the range of ADCs resulting from DNIF-processed images. Furthermore, ADCs are shown to be equivalent, as indicated by the relative difference of ADC means from NOA_9_ measurements (displayed as a percentage above NOA_1_ and NOA_1-DNIF_ violin plots).

After initial training of the denoising model on the 14 patients with prostate cancer, the model was assessed on the three patients in the validation dataset. An example of the DNIF being applied to each of the three validation patients from this sample (*b* = 900 sec/mm^2^ images and ADC maps) is illustrated in Figure 2; the DNIF was able to reduce the influence of imaging noise in the output image compared with the input NOA_1_ image, resulting in superior image quality in the subsequently calculated ADC maps. The NOA_1-DNIF_ images had improved quantitative metrics compared with the original NOA_1_ images for the MSE (5.8 × 10^−6^ vs 7.7 × 10^−6^; *P* < .001), SSIM (0.994 vs 0.992; *P* < .001), and PSNR (55.7 vs 53.2; *P* < .001) (Table 2). For all three validation patients within this sample, violin plots of ADCs within segmented regions demonstrated the ability of the DNIF model to reduce the range of calculated ADC measurements as a result of improving the SNR; the mean ADCs measured within bone disease from NOA_1-DNIF_ images deviated from the mean ADC calculated by using NOA_9_ images by an average RDM of 1.9% (range, 1.1%–2.6%) (within previously reported repeatability limits for mean ADC measurements [27]). The NOA_1-DNIF_ images also had a smaller average difference from the ground truth ADC coefficient of variation than did the NOA_1_ images (3.5% vs 9.0%), and the mean absolute voxel-wise difference was also smaller (123.4 vs 136.7). Detailed results are presented in Table 2.

**Table 2:**
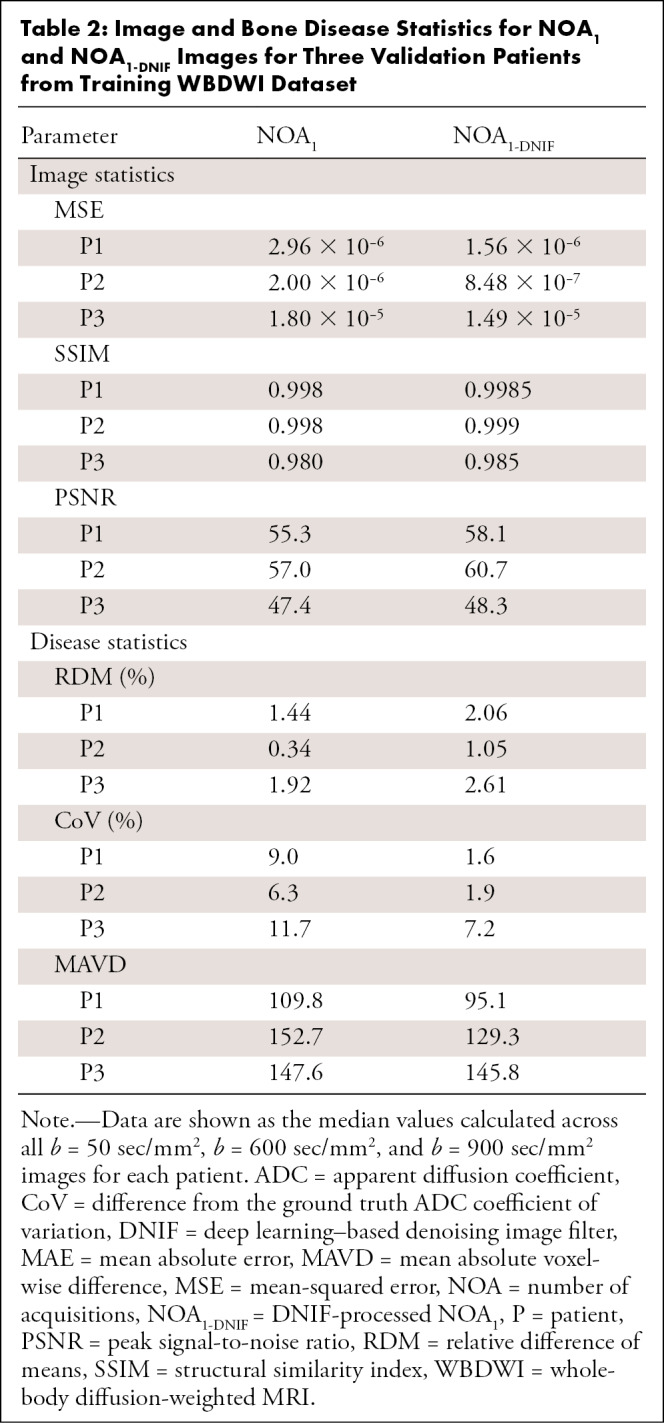
Image and Bone Disease Statistics for NOA_1_ and NOA_1-DNIF_ Images for Three Validation Patients from Training WBDWI Dataset

### Model Performance on the Test WBDWI Dataset

**Figure 3:**
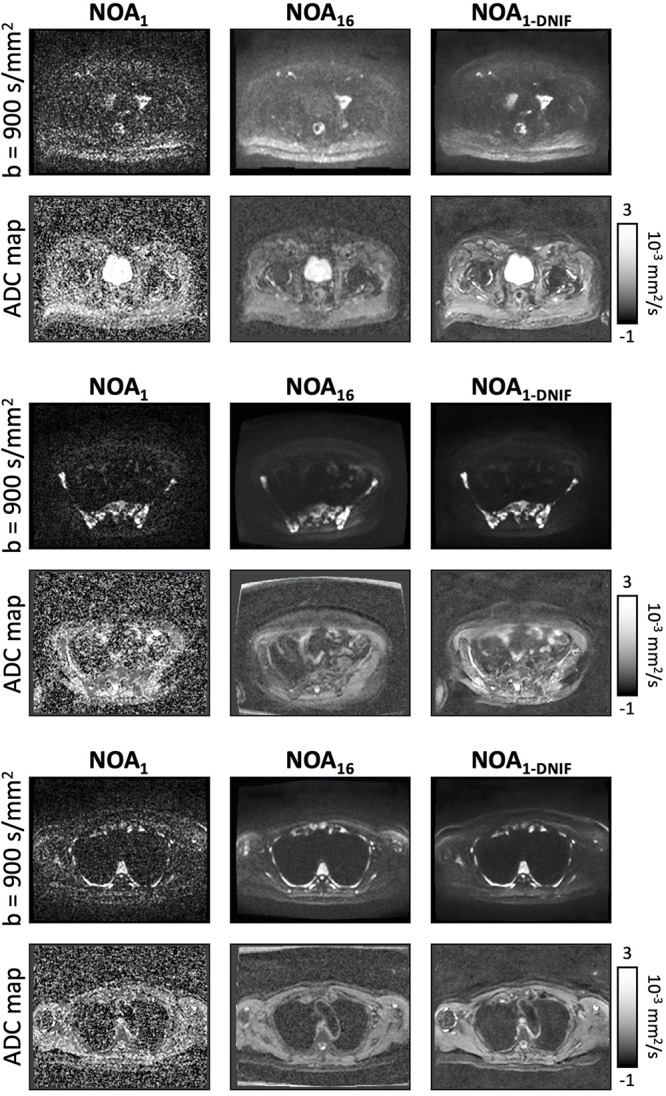
Example axial *b* = 900 sec/mm^2^ images and apparent diffusion coefficient (ADC) maps for the unfiltered (number of acquisitions = 1 [NOA_1_]) images, clinical-standard (NOA_16_) images, and deep learning–based denoising image filter (DNIF)-processed NOA_1_ (NOA_1-DNIF_) images for three of the patients in the test whole-body diffusion-weighted MRI dataset.

**Figure 4:**
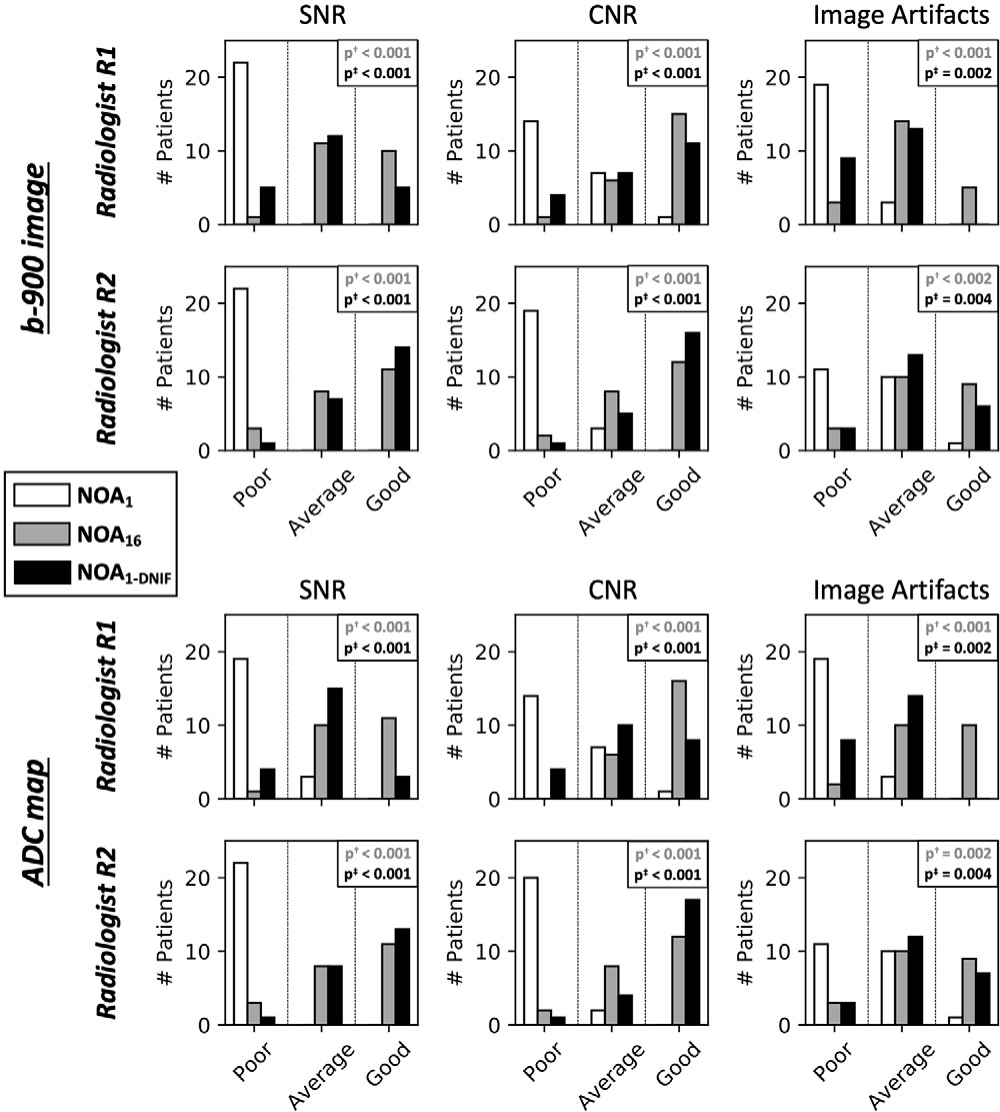
Bar plots for the observer rating study of the test whole-body diffusion-weighted MRI dataset for each image-quality criterion: the signal-to-noise ratio (SNR), the contrast-to-noise ratio (CNR), and image artifacts. Results are shown for *b* = 900 sec/mm^2^ images and apparent diffusion coefficient (ADC) maps separately. In all cases, the majority of fast-acquisition (number of acquisitions = 1 [NOA_1_]) datasets received a “poor” quality score for both *b* = 900 sec/mm^2^ images and ADC maps, whereas for the NOA_16_ dataset, the majority of cases received an “average” or “good” score. The use of the deep learning–based denoising image filter (DNIF) consistently increases the number of cases scoring as average or good for datasets obtained through just one acquisition. A significant difference in the image-quality scores is observed in all cases when comparing NOA_16_ images with NOA_1_ images and when comparing DNIF-processed NOA_1_ (NOA_1-DNIF_) images with NOA_1_ images. p^†^ = pairwise comparison of NOA_16_ scores minus NOA_1_ scores by two-tailed Wilcoxon signed rank test, p^‡^ = pairwise comparison of NOA_1-DNIF_ scores minus NOA_1_ scores by two-tailed Wilcoxon signed rank test.

The model was then assessed on a test dataset of 22 patients with advanced prostate cancer, advanced breast cancer, or myeloma-related bone disease. Application of the DNIF was successful in all patients. Visual improvements in image quality in terms of the contrast-to-noise ratio for high-*b*-value images and the resulting ADC maps were observed for all patients; results for six selected patients are illustrated in Figure 3, and examples from all patients are presented in Appendix E1 (supplement). Radiologist review of these images is summarized in Figure 4. The majority of NOA_1_ images (both *b* = 900 sec/mm^2^ images and ADC maps) were graded as “poor” by both radiologists across all quality criteria, whereas the majority of NOA_16_ and NOA_1-DNIF_ images were graded as either “average” or “good.” Statistically significant differences were observed in all comparisons (NOA_16_ vs NOA_1_ images and NOA_1-DNIF_ vs NOA_1_ images) for all quality metrics and for both radiologists independently. The average quality scores (± the standard error from the three-point quality scale) of the ADC maps obtained from NOA_1-DNIF_ images were higher than the scores of the ADC maps obtained from NOA_1_ images (SNR, 2.25 ± 0.10 vs 1.07 ± 0.04 [*P* < .005]; contrast-to-noise ratio, 2.45 ± 0.11 vs 1.25 ± 0.07 [*P* < .005]; image artifacts, 1.91 ± 0.1 vs 1.34 ± 0.08 [*P* < .005]). Table 3 presents the percentage of images defined to be clinically usable (average or good) by either radiologist; the majority of images were defined to be clinically usable for NOA_16_ and NOA_1-DNIF_ images, whereas this was not the case for NOA_1_ images.

**Table 3:**
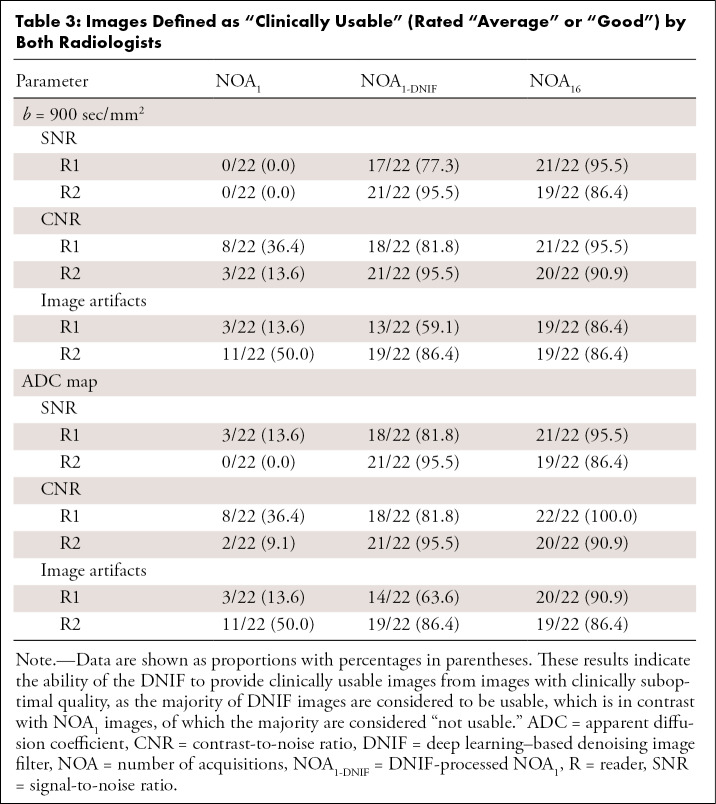
Images Defined as “Clinically Usable” (Rated “Average” or “Good”) by Both Radiologists****

### Performance of the Pretrained WBDWI and Retrained Lung Model on the Mesothelioma Dataset

**Figure 5:**
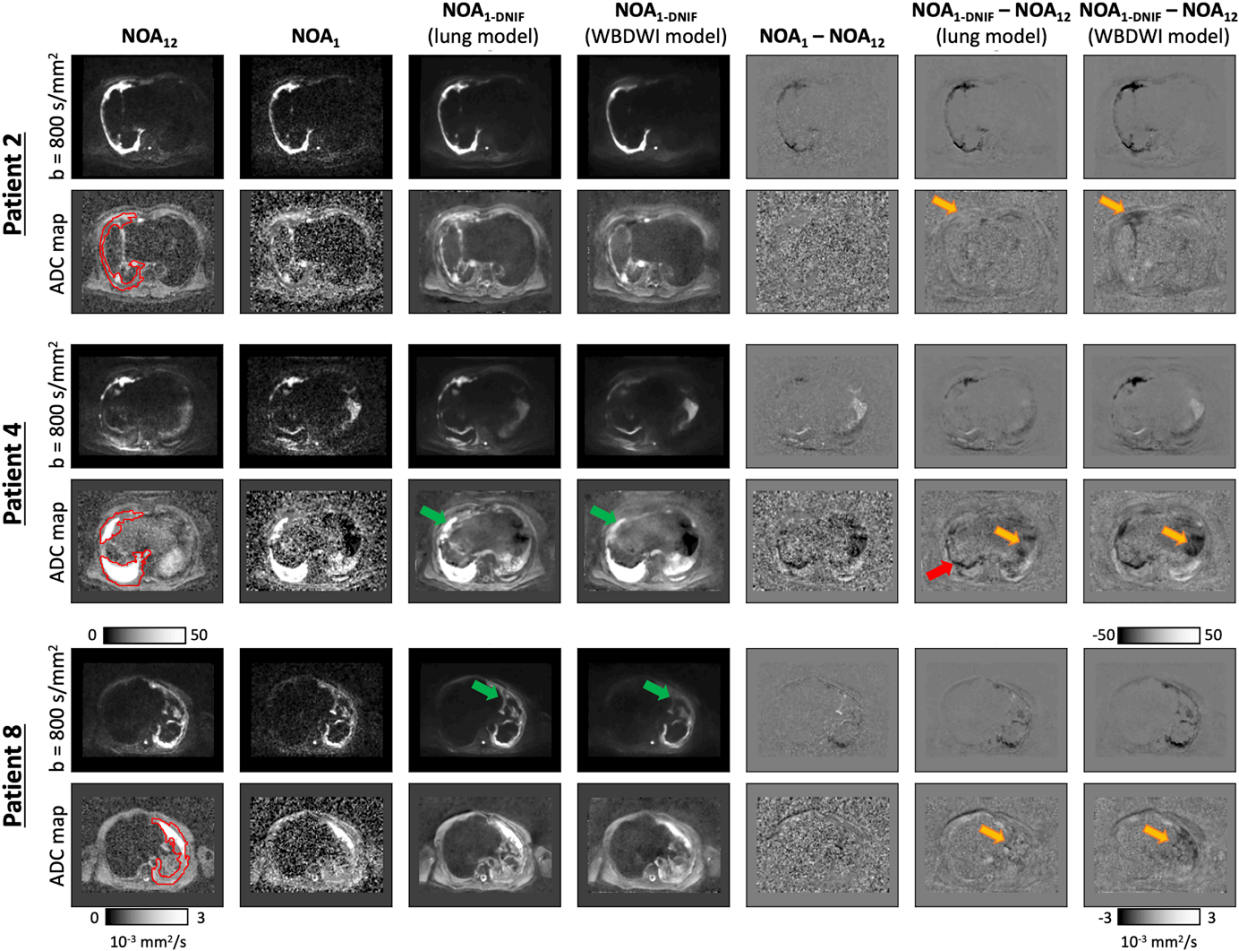
Three patient example datasets from the test arm of the mesothelioma dataset. High-*b*-value images (*b* = 800 sec/mm^2^: top row in each patient example) are displayed alongside apparent diffusion coefficient (ADC) maps (bottom row in each patient example) for the clinical-standard (number of acquisitions = 12 [NOA_12_]) images, the fast-acquisition (NOA_1_) images, and the deep learning–based denoising image filter (DNIF)-processed NOA_1_ (NOA_1-DNIF_) images from the pretrained whole-body diffusion-weighted MRI (WBDWI) model and the retrained lung model (which was retrained by using data acquired specifically in patients with malignant pleural mesothelioma). In addition, difference maps are shown between the NOA_12_ images and the NOA_1-DNIF_ or NOA_1_ images (NOA_1-DNIF_ − NOA_12_, for example). All equivalent images are displayed by using the same windowing settings. Although a clear improvement in image quality is observed when using the pretrained WBDWI, a further improvement is seen from the lung model. In particular, improved disease contrast can be observed in high-*b*-value images and ADC maps, with sharper tissue boundaries (green and orange arrows, respectively) being demonstrated. In a few cases, some bias is observed in the ADC calculations obtained by using the DNIF lung model (red arrow); this occurs in regions of motion (eg, near the diaphragm) where the NOA_12_ image signal will average out in regions that move (effective acquisition time on the order of minutes), whereas NOA_1_ images represent more of a snapshot in time (acquisition time on the order of tens of milliseconds).

**Figure 6:**
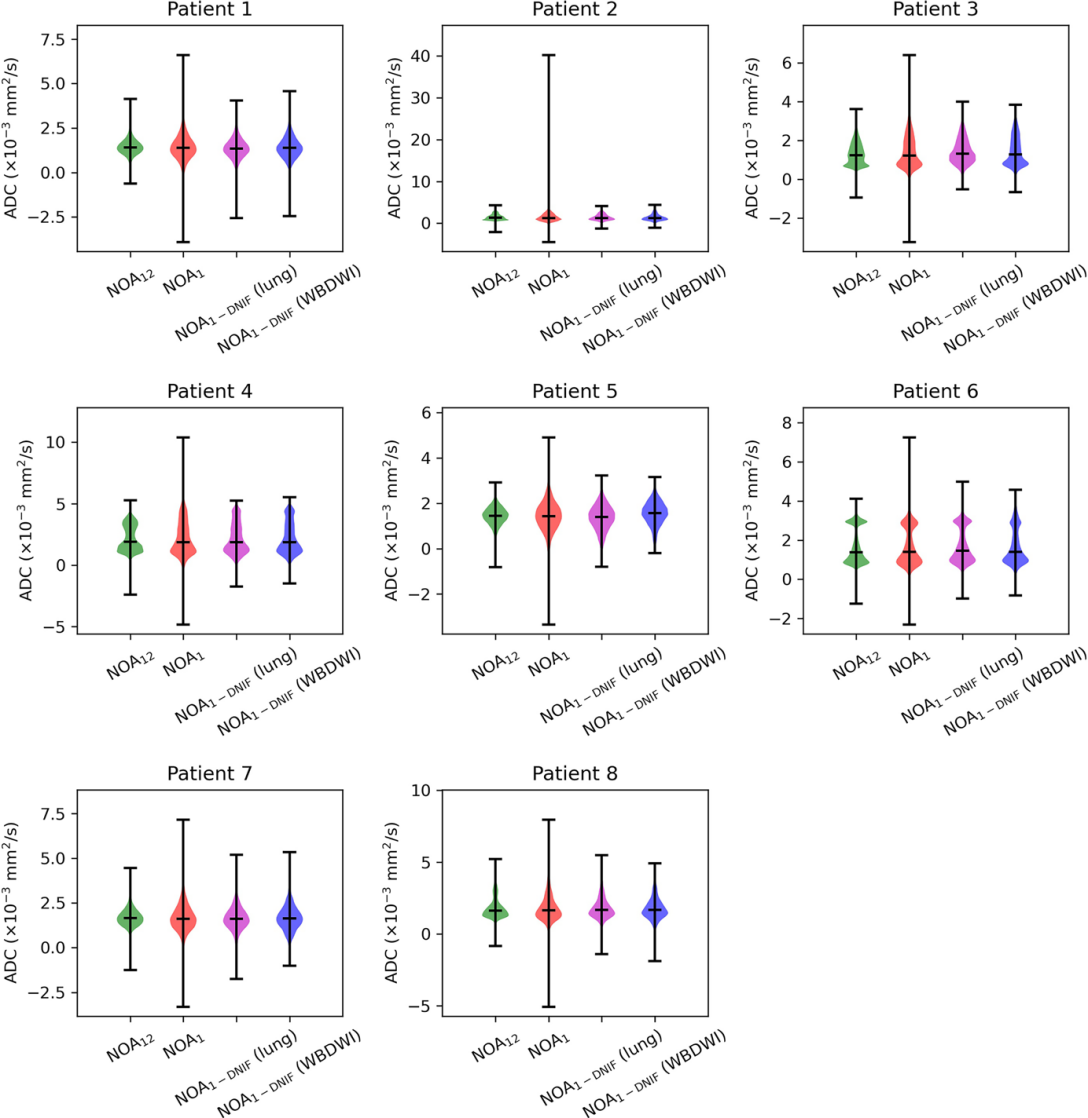
Violin plots of the apparent diffusion coefficient (ADC) distributions within segmented disease for all eight test patients in the mesothelioma dataset; example segmentation regions are displayed as red contours on the clinical-standard (number of acquisitions = 12 [NOA_12_]) ADC maps in Figure 5. Some differences were observed in these distributions, particularly at ADCs greater than 2 × 10^−3^ mm^2^/sec (patients 3 and 4, for example). Further investigation revealed that this was likely due to bulk motion, because NOA_1_ (and hence deep learning–based denoising image filter [DNIF]-processed NOA_1_ [NOA_1-DNIF_]) images are effectively snapshots in time (acquisition time on the order of tens of milliseconds), whereas the NOA_12_ image signal averages out motion over the 12 repeat measurements. In regions of pleural mesothelioma, where bulk free water flows as a result of convection from one imaging section to another, this could result in incomplete T1 relaxation of the water as it flows from one section to the next, leading to regions of spurious signal suppression on each section excitation. WBDWI = whole-body diffusion-weighted MRI.

Next, two different models were assessed on the mesothelioma dataset: the original pretrained WBDWI model and the model retrained on a subset of patients from the mesothelioma dataset (lung model). Figure 5 compares results for three of the validation patient datasets from the mesothelioma dataset, demonstrating NOA_1_ images filtered by using both the WBDWI model and the lung model. The lung model improved all three quantitative metrics (MSE, SSIM, and PSNR) in all eight test patients (Table 4). Analyzing the ADC distributions from all imaging techniques (Fig 6 and Table 4) revealed low RDM scores, with average values of 2.0% (range, 0.4%–8.4%) for NOA_1_ images and 3.7% (range, 0.2%–10.6%) and 4.0% (range, 0.1%–11.2%) for NOA_1-DNIF_ images derived from the lung model and the WBDWI model, respectively. In one patient (patient 3), the mean ADC of disease from NOA_1-DNIF_ images deviated from the mean ADC from NOA_12_ images by approximately 11%. However, a similar variation was observed for NOA_1_ ADC maps, indicating that this deviation was not due to the application of the DNIFs. In all cases, application of the DNIFs (NOA_1-DNIF_ images) reduced the presence of ADC measurement outliers in filtered images compared with NOA_1_ images. The NOA_1-DNIF_ ADC maps also had a smaller average difference from the ground truth ADC coefficient of variation and had a smaller mean voxel-wise difference in most cases (Table 4).

**Table 4:**
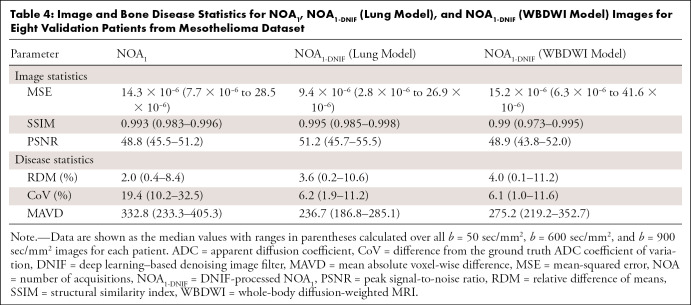
Image and Bone Disease Statistics for NOA_1_, NOA_1-DNIF_ (Lung Model), and NOA_1-DNIF_ (WBDWI Model) Images for Eight Validation Patients from Mesothelioma Dataset

## Discussion

Our DNIF improved image quality in subsampled WBDWI acquisitions as demonstrated within our test datasets of images from patients with metastatic prostate, breast, or myeloma-related bone disease. Initial results indicate that ADC measurements made by using DNIF-processed images fall within the typical limits of repeatability for mean extracranial ADC measurements (28) and are therefore comparable with those made by using fully sampled WBDWI images (in tumors for which isotropic water diffusion can be assumed). This indicates that DNIF-derived ADC estimates in bone disease might have a level of clinical image quality that is sufficient for monitoring the treatment response (26,29); repeat baseline measurements acquired by using our method would be required to fully test this hypothesis. In our blinded study based only on anatomic images from an independent set of 22 patients, two expert radiologists deemed the majority of DNIF-processed images as “usable” for the clinical setting, whereas the original noisy images from which they were derived were mostly “not usable.”

A major advantage of our approach is that the acquisition of training data needed for deriving the DNIF can be adopted by any imaging center, providing adaptable solutions that are trained to a particular manufacturer and/or imager. We have demonstrated that our method can be adapted to other diseases investigated by using DWI, such as MPM. Although the WBDWI-trained DNIF can be used to improve image quality of single-acquisition DWI images obtained in the context of MPM, the technique can be improved by acquiring disease-specific training data.

Understanding the inner workings of any deep learning algorithm is critical if such technologies are to be embraced in the health care sector, and this understanding is required to support application for medical regulatory approval. In Appendix E1 (supplement), we provide some evidence for how our DNIF may be working; we provide preliminary evidence that the DNIF is nonlinear, spatially variant, nonlocal, and edge preserving. We posit on the basis of these results that the DNIF is learning about the complex relationships among pixels within the image in terms of their relative position and relative intensity. Moreover, we suggest that the DNIF learns about anatomic position to tune the degree of smoothing it performs at a particular body location. This is evidenced by the improvements observed when retraining the DNIF for our MPM data; because of respiratory motion within the thoracic cage, the algorithm tended to oversmooth images in this region when using the WBDWI-trained DNIF.

During training, the neural network minimizes a cost function that measures the similarity between the DNIF-processed images and the clinical-standard images used as the ground truth. The correct assessment of image similarity by algorithms is an ongoing problem in the computer vision field. The default choice, the MSE, is predominantly used for its simplicity and well-understood properties but has limitations, including the assumption that noise has a Gaussian distribution and is not dependent on local image characteristics (30). Furthermore, this metric, although valid for other applications, produces images that do not correlate well with human perception of image quality (two images with a very low MSE can look quite different to a human observer) (24). In this study, we investigated the MAE and combined it with a metric that can be used to more closely resemble human perception, the SSIM (25). In our future studies, we aim to further explore other approaches, such as the use of a perceptual loss (as deep features have been shown to correlate better with human perception than do manual metrics [31,32]) and generative adversarial network architectures (33), while also comparing these approaches with traditional denoising algorithms (34,35).

The encouraging findings of our proof-of-concept study warrant further investigation through multicenter studies comprising larger patient populations to understand the effect of the technique on diagnostic accuracy. Deep neural networks typically benefit from the addition of training data from other institutions, MRI vendors, and different protocols and would offer a filter that produces images that are of clinical quality such that it would enable evaluation of any WBDWI study. Our approach could exploit the concept of “transfer learning.” By using the weights from our DNIF as an initialization, an individual site may not need to acquire much data to train a network specific to that site. Future studies could also investigate the value of working directly with acquired raw k-space data for improving single-shot WBDWI image quality by using contemporary methods in machine learning, such as Automated Transform by Manifold Approximation (36,37). In a few patients, we found some differences between the calculated ADCs from DNIF images and the calculated ADCs from clinical images, especially for images acquired at *b* values greater than 2 × 10^−3^ mm^2^/sec. This appears to be due to the fact that the DNIF images capture a snapshot in time (tens of milliseconds per *b*-value image), whereas the clinical images comprise an average of nine or 12 repeat acquisitions obtained over approximately 5 minutes, thus averaging out motion effects. In some respects, this is encouraging, because it warrants further exploration of the use of DNIF for fast-acquisition, breath-hold ADC measurements in the abdomen and chest.

We conclude that deep learning methods, such as our quickDWI approach, are able to improve the quality of WBDWI images from subsampled data, potentially reducing acquisition times by a significant amount (from approximately 25 minutes to 5 minutes in our test study). Such time savings would reduce imaging costs, rendering WBDWI appropriate for screening studies and reducing patient imaging time and/or discomfort, which could aid in the widespread adoption of WBDWI.
